# Facilitation of phosphorus uptake in maize plants by mycorrhizosphere bacteria

**DOI:** 10.1038/s41598-017-04959-0

**Published:** 2017-07-05

**Authors:** Fabio Battini, Mette Grønlund, Monica Agnolucci, Manuela Giovannetti, Iver Jakobsen

**Affiliations:** 1 Department of Agriculture, Food and Environment, University of Pisa, Pisa, 56124 Italy; 2 Department of Chemical and Biochemical Engineering, Technical University of Denmark, Lyngby, DK-2800 Kgs. Denmark; 3 Department of Plant and Environmental Sciences, Faculty of Science, University of Copenhagen, 1871 Frederiksberg C, Copenhagen, Denmark

## Abstract

A major challenge for agriculture is to provide sufficient plant nutrients such as phosphorus (P) to meet the global food demand. The sufficiency of P is a concern because of it’s essential role in plant growth, the finite availability of P-rock for fertilizer production and the poor plant availability of soil P. This study investigated whether biofertilizers and bioenhancers, such as arbuscular mycorrhizal fungi (AMF) and their associated bacteria could enhance growth and P uptake in maize. Plants were grown with or without mycorrhizas in compartmented pots with radioactive P tracers and were inoculated with each of 10 selected bacteria isolated from AMF spores. Root colonization by AMF produced large plant growth responses, while seven bacterial strains further facilitated root growth and P uptake by promoting the development of AMF extraradical mycelium. Among the tested strains, *Streptomyces* sp. W94 produced the largest increases in uptake and translocation of ^33^P, while *Streptomyces* sp. W77 highly enhanced hyphal length specific uptake of ^33^P. The positive relationship between AMF-mediated P absorption and shoot P content was significantly influenced by the bacteria inoculants and such results emphasize the potential importance of managing both AMF and their microbiota for improving P acquisition by crops.

## Introduction

In the next decades, a major challenge for agriculture will be the sustainable production of enough food crops to meet the growing global demand. Current agricultural systems strongly depend on continuous applications of chemical fertilizers, mainly nitrogen (N), phosphorus (P) and potassium (K), which contribute to the decline of biological soil fertility^1–3^. In particular, the use of phosphate-based fertilizers has increased from approximately 5 million tons P per year in 1961 to approximately 20 million tons in 2013^4^. Phosphorus is required in relatively large amounts by plants for optimal growth, as it is a structural constituent of biomolecules involved in several key plant processes, including photosynthesis, nucleic acid and phospholipid biosynthesis, respiration and energy transfer^5, 6^. Most agricultural soils contain high concentrations of P, in both inorganic and organic forms, that is poorly available for plant uptake due to its immobilization and precipitation with other soil minerals, such as iron (Fe) and aluminium (Al) in acid soils and calcium (Ca) in alkaline soils. Thus, only a small percentage of P (<1%) is directly available for plant uptake^7, 8^. The abundant plant root-associated microbiota represents one sustainable strategy for the exploitation and mobilization of the soil P pool^9^.

Arbuscular mycorrhizal (AM) fungi (AMF, *Glomeromycota*) are important beneficial soil microorganisms establishing mutualistic associations with most food crops. These associations increase plant nutrient uptake and tolerance to root pathogens and drought. AMF are obligate biotrophs and colonize host roots to obtain sugars in exchange of mineral nutrients, absorbed and translocated through a fine network of extraradical mycelium (ERM) spreading from colonized roots into the soil^10^. Such belowground networks, which show length densities, up to about 25 m g^−1^ soil^11, 12^, function as auxiliary absorbing systems that transfer mineral nutrients, such as phosphorus (P), nitrogen (N), sulfur (S), potassium (K), calcium (Ca), iron (Fe), copper (Cu), and zinc (Zn), from the soil outside the roots to the host plants^13–16^. AMF mycorrhizal networks may differentially affect P and N supply to host plants depending on their structural and functional properties^17–20^, including the occurrence and differential expression of P transporter and N assimilation fungal genes^21–23^. The improved P nutrition of mycorrhizal plants is also the result of hyphal P uptake beyond the P depletion zone caused by the fast root absorption of P from the soil solution, which cannot be rapidly replenished, given the poor mobility of P in the soil^8, 10^. The P taken up by ERM is translocated to intraradical hyphae in the form of polyphosphates^24–26^. High P fluxes in AMF hyphae have been detected, ranging from 2 to 20 × 10^−6^ mol m^−2^ s^−1^ ^27–29^, with bidirectional protoplasmic flows, measured on the basis of cellular particles movement (vacuoles, nuclei, fat droplets, organelles, granules), ranging from 3.0 to 4.3 μm s^−1^ ^30, 31^.

The development and performance of AMF may be mediated by a third component of the symbiosis, represented by highly diverse bacterial communities living associated with AMF spores and mycelium (mycorrhizospheric microbiota)^32–36^. Such beneficial bacteria showed key plant growth promoting (PGP) functions, encompassing nitrogen fixation, P solubilization, the production of indole acetic acid (IAA), siderophores and antibiotics, supplying fundamental nutrients and growth factors^37–42^. Recently, a culture-dependent approach allowed the isolation in pure culture of 374 bacterial strains strictly associated with *Rhizophagus irregularis* (formerly *Glomus intraradices*) spores, and the selection of Actinobacteria, Bacillaceae and *Sinorhizobium meliloti* strains showing several PGP activities, including P mineralization from phytate and mineral P solubilization^43^.

This work, for the first time, investigated to which degree growth and P uptake of mycorrhizal and non-mycorrhizal maize plants would benefit from inoculation with a selection of bacterial strains isolated from AMF spores, showing multiple PGP activities *in vitro*. *Bacillus* spp., *Sinorhizobium meliloti* and *Streptomyces* spp. were inoculated alone or in combination with *R. irregularis* in two experiments using compartmented pots with a root hyphal compartment (RHC) and a hyphal compartment (HC). Labelling of HC soil with radioactive P (^32^P or ^33^P) made it possible to distinguish between P uptake by AM hyphae and P uptake by roots and hyphae in combination. While AM colonization resulted in large plant growth responses, the bacteria inoculants increased the abundance of AMF hyphae in soil and four of them also root P content and dry weight.

## Results

### Root growth and AMF abundance in roots and hyphal compartment soil

Here we report data on total and colonized root length of maize plants grown in the RHC and on hyphal length occurring in the HC, obtained from two successive experiments where the PGP bacterial strains (3 strains in Exp. 1, 8 strains in Exp. 2) were inoculated alone and in combination with the AM fungus *R. irregularis*. Roots of AMF inoculated plants were well colonized in both experiments and had a distinct yellow-orange root colour due to apocarotenoid accumulation^44^, while roots of non-inoculated plants remained uncolonized and pale. Bacterial inoculation had no effects on mycorrhizal colonization, which ranged from 78% to 87% in Exp. 1, and from 58 to 67% in Exp. 2 (Table 1). The root length was strongly and consistently increased by AM colonization, in particular in Exp. 2. Maize plants inoculated with *R. irregularis* alone or in combination with bacteria, showed root length increases of 62 and 211% in Exp. 1 and in Exp. 2, respectively, compared with non-mycorrhizal (NM) controls (Table 1).

**Table 1:** Mycorrhizal colonization, root length, colonized root length and hyphal length density in HC soil in *Zea mays* L. Oh43 inoculated, or not (NM), with *Rhizophagus irregularis* (AMF) alone or in combination with different bacterial strains.

Inoculum type	Mycorrhizal colonization (%)	Root length (m)	Colonized root length (m)	Hyphal length density (m g^−1^)
**Experiment 1**				
NM	0	81 ± 8.3 a	0	0
NM + *S. meliloti* TSA3^1^	0	91 ± 11.7 ab	0	0
NM + *S. meliloti* TSA41	0	112 ± 11.4 abc	0	0
NM + *L. fusiformis* CH19	0	112 ± 8.6 abc	0	0
AMF	80 ± 2.1	148 ± 8.7 abc	118 ± 5.9	22.3 ± 0.61 a
AMF + *S. meliloti* TSA3^1^	78 ± 3.3	163 ± 28.5 bc	130 ± 25.4	22.5 ± 0.80 a
AMF + *S. meliloti* TSA41^2^	87 ± 1.2	159 ± 10.2 bc	137 ± 8.2	26.5 ± 1.16 b
AMF + *L. fusiformis* CH19	81 ± 3.3	173 ± 27.7 c	139 ± 20.4	24.6 ± 0.90 ab
**One-way ANOVA (P values)**	0.355	**0.002**	0.846	**0.020**
**Experiment 2**				
NM	0	91 ± 11.9 a	0	0
NM + *S. meliloti* TSA41	0	123 ± 13.2 a	0	0
NM + *S. meliloti* TSA26	0	121 ± 10.0 a	0	0
NM + *Streptomyces* sp. W43N	0	114 ± 21.7 a	0	0
NM + *Streptomyces* sp. W64^1^	0	69 ± 12.9 a	0	0
NM + *Streptomyces* sp. W77^1^	0	129 ± 17.3 a	0	0
NM + *Streptomyces* sp. W94	0	121 ± 20.5 a	0	0
NM + *S. meliloti* N29^2^	0	70 ± 18.8 a	0	0
NM + *Bacillus* sp. CH10	0	139 ± 11.6 a	0	0
AMF	62 ± 2.4	318 ± 13.2 b	197 ± 13.3	21.6 ± 0.41 a
AMF + *S. meliloti* TSA41	60 ± 2.6	334 ± 23.9 b	201 ± 17.9	26.9 ± 0.46 c
AMF + *S. meliloti* TSA26	67 ± 2.3	316 ± 34.3 b	211 ± 22.5	26.6 ± 0.28 c
AMF + *Streptomyces* sp. W43N	58 ± 4.2	383 ± 9.8 b	221 ± 15.5	26.9 ± 0.44 c
AMF + *Streptomyces* sp. W64	62 ± 3.2	354 ± 38.6 b	218 ± 25.5	26.6 ± 0.63 c
AMF + *Streptomyces* sp. W77	58 ± 1.9	374 ± 21.3 b	218 ± 13.8	22.4 ± 0.42 ab
AMF + *Streptomyces* sp. W94	58 ± 1.8	355 ± 11.9 b	208 ± 11.4	26.2 ± 0.50 c
AMF + *S. meliloti* N29	59 ± 1.7	285 ± 31.4 b	168 ± 17.7	26.2 ± 0.60 c
AMF + *Bacillus* sp. CH10	61 ± 3.9	315 ± 41.0 b	193 ± 28.7	25.0 ± 0.52 bc
**One-way ANOVA (** ***P*** **values)**	0.397	**<0.001**	0.605	**<0.001**

Data are presented as the mean ± s.e.m. (n = 5). Different letters indicate significantly different values based on one-way ANOVA (*P* < 0.05). *Values in bold type indicate factors that had significant effects. ^1^Treatment with four biological replicates; ^2^Treatment with three biological replicates.

The extraradical mycelium, but not roots, proliferated into all HCs of AMF-inoculated pots and the measured hyphal length densities (HLD) were corrected by subtraction of the average background values measured in HCs of NM treatments (0.15 ± 0.04–0.90 ± 0.10 m g^−1^ soil). Inoculation with bacteria increased HLD significantly in both experiments (Exp. 1, *P* = 0.020; Exp. 2, *P* < 0.001; Table 1). This stimulation of HLD was caused by *S. meliloti* TSA41 in Exp. 1 and by all bacteria except *Streptomyces* sp. W77 in Exp. 2. Interestingly, *S. meliloti* TSA41 was the most stimulatory bacterium in both Exp. 1 and 2, where HLD increased by 19 and 25% over the levels measured in mycorrhizal plants without bacterial inoculation.

### P content and P uptake

**Figure 1:**
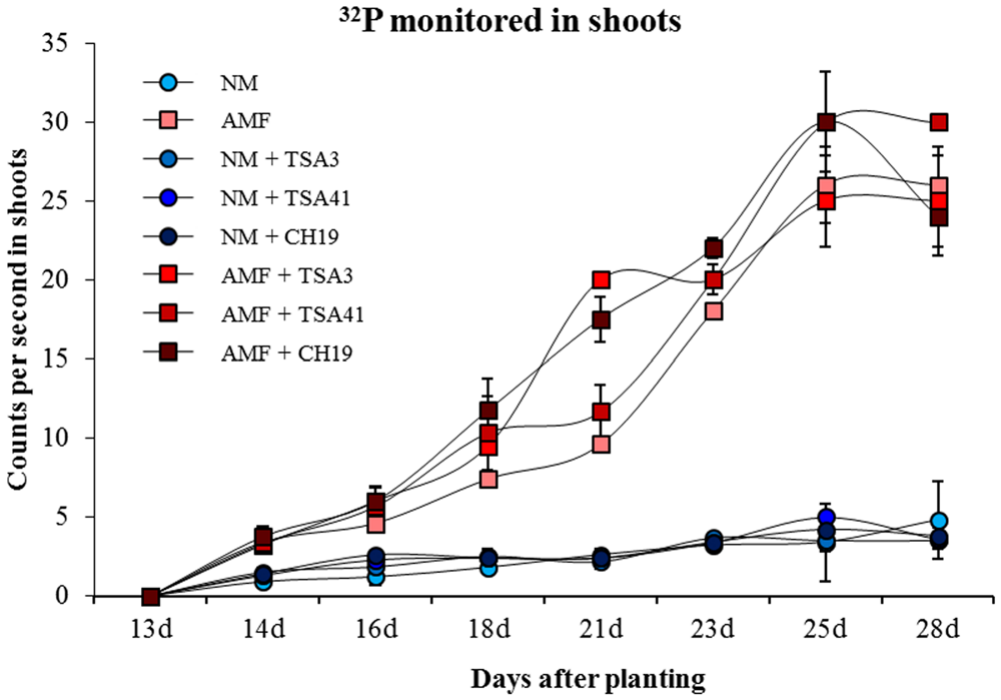
Time course of radioactivity (^32^P, counts per second) monitored in the shoots of *Zea mays* L. genotype Oh43 inoculated (red squares) or not (blue circles) with *Rhizophagus irregularis* alone or in combination with different bacterial strains, in Exp. 1. TSA3: *Sinorhizobium meliloti* TSA3; TSA41: *Sinorhizobium meliloti* TSA41; CH19: *Lysinibacillus fusiformis* CH19. Bars represent standard errors.

Here, data are reported on P uptake by AM hyphae and by roots and hyphae in combination, as revealed by the use of radioactive P (^32^P or ^33^P) added to HC soil. In Exp. 1, radioactivity (^32^P) was consistently detected in shoots already 14 days after planting (Fig. 1). Even though the monitoring allowed for only semi-quantitative measurement of radioactivity, the ^32^P content in shoots differed clearly between plants inoculated with *R. irregularis* and uninoculated controls. Moreover, mycorrhizal plants showed higher counts when co-inoculated with bacteria than when non-co-inoculated, at four early time points (16, 18, 21 and 23d).

In Exp. 1, shoot and root P content, and root length specific P uptake were 177%, 196% and 80% greater in mycorrhizal than in NM treatments (*P* < 0.001) (Table 2). In Exp. 2, as AMF inoculation strongly increased plant variables, not allowing the satisfaction of data normality and homogeneity of variances, values of mycorrhizal and NM plants were analysed separately by one-way ANOVA, followed by Student’s *t* test to compare the pooled means (Table 2). As expected, shoot and root P content, as well as root specific P uptake of maize plants responded strongly to mycorrhizal colonization (*P* < 0.001). Bacterial inoculation of NM plants significantly increased root P content (*P* = 0.036), with increases higher than 80% in plants inoculated with *Streptomyces* sp. W43N and W94, *Bacillus* sp. CH10 and *S. meliloti* TSA26. In contrast, root length specific P uptake was not influenced by bacterial inoculation (*P* = 0.087). In mycorrhizal plants, inoculation with *Streptomyces* sp. W94 significantly increased ^33^P content in shoots (*P* < 0.001), compared with the corresponding control. Bacterial inoculation significantly increased hyphal length specific ^33^P uptake (*P* < 0.001) by 37 and 39% in plants inoculated with *Streptomyces* sp. W94 and W77, respectively, while it decreased by 28% in plants inoculated with *S. meliloti* N29.

**Table 2:** P content and P uptake of *Zea mays* L. genotype Oh43 inoculated, or not (NM), with *Rhizophagus irregularis* (AMF) alone or in combination with different bacterial strains.

Inoculum type	Shoot P content (mg plant^−1^)	Root P content (mg plant^−1^)	Root length specific P uptake (µg m^−1^)	Shoot ^32^P or ^33^P (kBq plant^−1^)	Hyphal length specific ^33^P/^32^P uptake (Bq m^−1^)
**Experiment 1**					
NM	3.13 ± 0.81 a	0.91 ± 0.09 a	49 ± 7 abcd	—	—
NM + *S. meliloti* TSA3^1^	2.65 ± 0.13 a	0.80 ± 0.07 a	39 ± 4 a	—	—
NM + *S. meliloti* TSA41	3.39 ± 0.22 a	1.09 ± 0.08 a	41 ± 3 ab	—	—
NM + *L. fusiformis* CH19	3.23 ± 0.15 a	1.64 ± 0.36 a	45 ± 5 abc	—	—
AMF	8.41 ± 0.20 b	3.04 ± 0.09 b	78 ± 5 bcd	32.1 ± 0.83	26.31 ± 0.94
AMF + *S. meliloti* TSA3^1^	8.54 ± 0.23 b	3.10 ± 0.49 b	83 ± 20 c	38.2 ± 3.68	31.03 ± 3.33
AMF + *S. meliloti* TSA41^2^	8.84 ± 0.14 b	3.60 ± 0.34 b	79 ± 5 cd	41.0 ± 1.92	28.32 ± 1.84
AMF + *L. fusiformis* CH19	8.63 ± 0.49 b	3.45 ± 0.31 b	73 ± 7 abcd	32.9 ± 2.92	25.40 ± 1.45
**One—way ANOVA (** ***P*** **values)**	**<0.001**	**<0.001**	**0.001**	0.180	0.227
**Experiment 2**					
NM	2.12 ± 0.23	0.70 ± 0.19 a	32 ± 3	—	—
NM + *S. meliloti* TSA41	2.94 ± 0.17	1.10 ± 0.14 ab	34 ± 3	—	—
NM + *S. meliloti* TSA26	2.52 ± 0.41	1.28 ± 0.15 b	31 ± 2	—	—
NM + *Streptomyces* sp. W43N	2.36 ± 0.35	1.30 ± 0.14 b	34 ± 3	—	—
NM + *Streptomyces* sp. W64^1^	1.76 ± 0.21	0.60 ± 0.12 a	36 ± 2	—	—
NM + *Streptomyces* sp. W77^1^	2.50 ± 0.34	1.18 ± 0.27 ab	28 ± 0	—	—
NM + *Streptomyces* sp. W94	2.92 ± 0.23	1.27 ± 0.25 b	36 ± 3	—	—
NM + *S. meliloti* N29	2.52 ± 0.22	0.88 ± 0.17 ab	34 ± 3	—	—
NM + *Bacillus* sp. CH10	2.76 ± 0.16	1.34 ± 0.09 b	30 ± 1	—	—
**One-way ANOVA (** ***P*** **values)**	0.105	**0.036**	0.087	—	—
AMF	9.11 ± 0.27	6.03 ± 0.29	48 ± 1	14.7 ± 1.76 ab	12.38 ± 1.33 b
AMF + *S. meliloti* TSA41	8.19 ± 0.78	5.76 ± 0.52	42 ± 3	17.0 ± 1.22 abc	11.51 ± 0.93 ab
AMF + *S. meliloti* TSA26	7.88 ± 0.53	5.48 ± 0.35	44 ± 2	16.0 ± 1.80 ab	10.92 ± 1.16 ab
AMF + *Streptomyces* sp. W43N	8.81 ± 0.30	7.05 ± 0.54	41 ± 2	18.7 ± 1.07 abc	12.72 ± 0.88 b
AMF + *Streptomyces* sp. W64	8.16 ± 0.31	6.55 ± 0.53	43 ± 4	15.9 ± 1.02 ab	10.93 ± 0.75 ab
AMF + *Streptomyces* sp. W77	8.58 ± 0.33	7.10 ± 0.75	42 ± 2	21.1 ± 1.96 bc	17.16 ± 1.64 c
AMF + *Streptomyces* sp. W94	8.63 ± 0.43	6.71 ± 0.57	43 ± 2	24.3 ± 2.54 c	16.91 ± 1.94 c
AMF + *S. meliloti* N29	7.20 ± 0.52	5.22 ± 0.41	45 ± 2	12.0 ± 1.19 a	8.41 ± 0.97 a
AMF + *Bacillus* sp. CH10	8.00 ± 0.24	5.98 ± 0.40	46 ± 4	14.8 ± 1.28 ab	10.94 ± 1.26 ab
**One-way ANOVA (P values)**	0.151	0.108	0.843	**<0.001**	**<0.001**
*t* test (*P* values) NM vs AMF	**<0.001**	**<0.001**	**0.002**	—	—

Data are presented as the mean ± s.e.m. (n = 5). Different letters indicate significantly different values (*P* < 0.05). As AMF inoculation strongly increased plant variables, not allowing the satisfaction of data normality and homogeneity of variances, values of mycorrhizal and NM plants were analysed separately by one-way ANOVA, followed by Student’s *t* test to compare the pooled means. *Values in bold type indicate factors that had significant effects. ^1^Treatment with four biological replicates; ^2^Treatment with three biological replicates.

### Growth responses

Here we report data on plant height, stem diameter, shoot dry weight (DW) and root DW of maize plants grown for 30 days in the experimental trials. In Exp. 1, stem diameter and shoot DW were consistently higher in mycorrhizal plants than in non-mycorrhizal controls, either inoculated or not-inoculated with bacteria. Variable responses were obtained for height and root DW (Table 3). In Exp. 2, as AMF inoculation strongly increased plant variables, not allowing the satisfaction of data normality and homogeneity of variances, values of mycorrhizal and control plants were analysed separately by one-way ANOVA, followed by Student’s *t* test to compare the pooled means (Table 3). Shoot and root DW, as well as maize plants height, responded strongly to AMF colonization (*P* < 0.001) (Table 3). In NM plants, neither shoot DW (*P* = 0.055) nor root DW (*P* = 0.069) were significantly influenced by bacterial inoculation (Table 3). In AM-colonized plants, shoot DW (*P* = 0.016) and root DW (*P* = 0.012) were significantly affected by bacterial inoculation, depending on the strains. The highest shoot DW was found in plants inoculated with *Streptomyces* sp. W77, which significantly differed from shoot DW of *S. meliloti* TSA41 and *S. meliloti* N29 inoculated plants. Inoculation with *Streptomyces* sp. W43N was the only treatment producing a significantly higher root DW, compared with AMF control plants (Table 3).

**Table 3:** Growth responses of *Zea mays* L. genotype Oh43 inoculated, or not (NM), with *Rhizophagus irregularis* (AMF) alone or in combination with different bacterial strains.

Inoculum type	Height (cm)	Stem diameter (cm)	Shoot DW (g plant^−1^)	Root DW (g plant^−1^)
**Experiment 1**				
NM	61.7 ± 1.68 a	0.92 ± 0.03 a	2.08 ± 0.34 a	0.93 ± 0.10 a
NM + *S. meliloti* TSA3^1^	63.8 ± 4.82 ab	1.07 ± 0.01 a	2.10 ± 0.37 a	0.97 ± 0.13 a
NM + *S. meliloti* TSA41	68.8 ± 1.48 ab	1.06 ± 0.04 a	2.81 ± 0.21 a	1.39 ± 0.07 ab
NM + *L. fusiformis* CH19	70.5 ± 1.21 ab	1.07 ± 0.03 a	2.89 ± 0.15 a	1.47 ± 0.11 ab
AMF	72.7 ± 0.62 b	1.31 ± 0.04 bc	4.37 ± 0.08 b	2.06 ± 0.10 bc
AMF + *S. meliloti* TSA3^1^	71.4 ± 0.51 ab	1.30 ± 0.05 bc	4.43 ± 0.38 b	2.32 ± 0.40 bc
AMF + *S. meliloti* TSA41^2^	71.3 ± 2.64 ab	1.47 ± 0.05 c	4.73 ± 0.14 b	2.60 ± 0.12 c
AMF + *L. fusiformis* CH19	73.8 ± 2.85 b	1.25 ± 0.04 b	4.51 ± 0.46 b	2.12 ± 0.34 bc
**One-way ANOVA (P values)**	**0.004**	**<0.001**	**<0.001**	**<0.001**
**Experiment 2**				
NM	59.8 ± 2.93 b	—	1.56 ± 0.18	0.90 ± 0.16
NM + *S. meliloti* TSA41	65.4 ± 1.93 b	—	2.07 ± 0.12	1.48 ± 0.20
NM + *S. meliloti* TSA26	62.3 ± 2.11 b	—	1.89 ± 0.21	1.38 ± 0.20
NM + *Streptomyces* sp. W43N	59.2 ± 3.63 b	—	1.75 ± 0.26	1.32 ± 0.23
NM + *Streptomyces* sp. W64^1^	52.4 ± 1.91 a	—	1.16 ± 0.19	0.62 ± 0.12
NM + *Streptomyces* sp. W77^1^	61.3 ± 3.33 b	—	1.76 ± 0.25	1.36 ± 0.26
NM + *Streptomyces* sp. W94	64.8 ± 1.28 b	—	2.18 ± 0.18	1.61 ± 0.32
NM + *S. meliloti* N29	62.5 ± 1.94 b	—	1.97 ± 0.19	0.62 ± 0.28
NM + *Bacillus* sp CH10	62.4 ± 2.01 b	—	1.90 ± 0.16	1.48 ± 0.17
**One-way ANOVA (** ***P*** **values)**	0.045*	—	0.055	0.069
AMF	71.2 ± 0.63	—	5.93 ± 0.22 bc	4.97 ± 0.51 abc
AMF + *S. meliloti* TSA41	70.2 ± 0.94	—	5.63 ± 0.17 ab	4.44 ± 0.28 ab
AMF + *S. meliloti* TSA26	73.5 ± 1.65	—	6.13 ± 0.59 bc	4.38 ± 0.49 ab
AMF + *Streptomyces* sp. W43N	72.3 ± 0.58	—	6.39 ± 0.19 bc	6.55 ± 0.54 d
AMF + *Streptomyces* sp. W64	69.1 ± 1.59	—	6.01 ± 0.42 bc	6.16 ± 0.86 cd
AMF + *Streptomyces* sp. W77	72.3 ± 1.55	—	6.72 ± 0.16 c	5.84 ± 0.55 bcd
AMF + *Streptomyces* sp. W94	71.9 ± 0.95	—	6.52 ± 0.24 bc	5.40 ± 0.32 bcd
AMF + *S. meliloti* N29	67.8 ± 1.39	—	4.64 ± 0.54 a	3.82 ± 0.67 a
AMF + *Bacillus* sp CH10	70.5 ± 1.27	—	5.83 ± 0.42 bc	4.43 ± 0.43 ab
**One-way ANOVA (** ***P*** **values)**	0.069	—	**0.016**	**0.012**
***t*** **test (** ***P*** **values) NM vs AMF**	**<0.001**	—	**<0.001**	**<0.001**

Data are presented as the mean ± s.e.m. (n = 5). Different letters indicate significantly different values (*P* < 0.05). As AMF inoculation strongly increased plant variables, not allowing the satisfaction of data normality and homogeneity of variances, values of mycorrhizal and NM plants were analysed separately by one-way ANOVA, followed by Student’s *t* test to compare the pooled means. *Values in bold type indicate factors that had significant effects. ^1^Treatment with four biological replicates; ^2^Treatment with three biological replicates.

### Relationships between plant growth and P uptake as influenced by microbial inoculants

**Figure 2:**
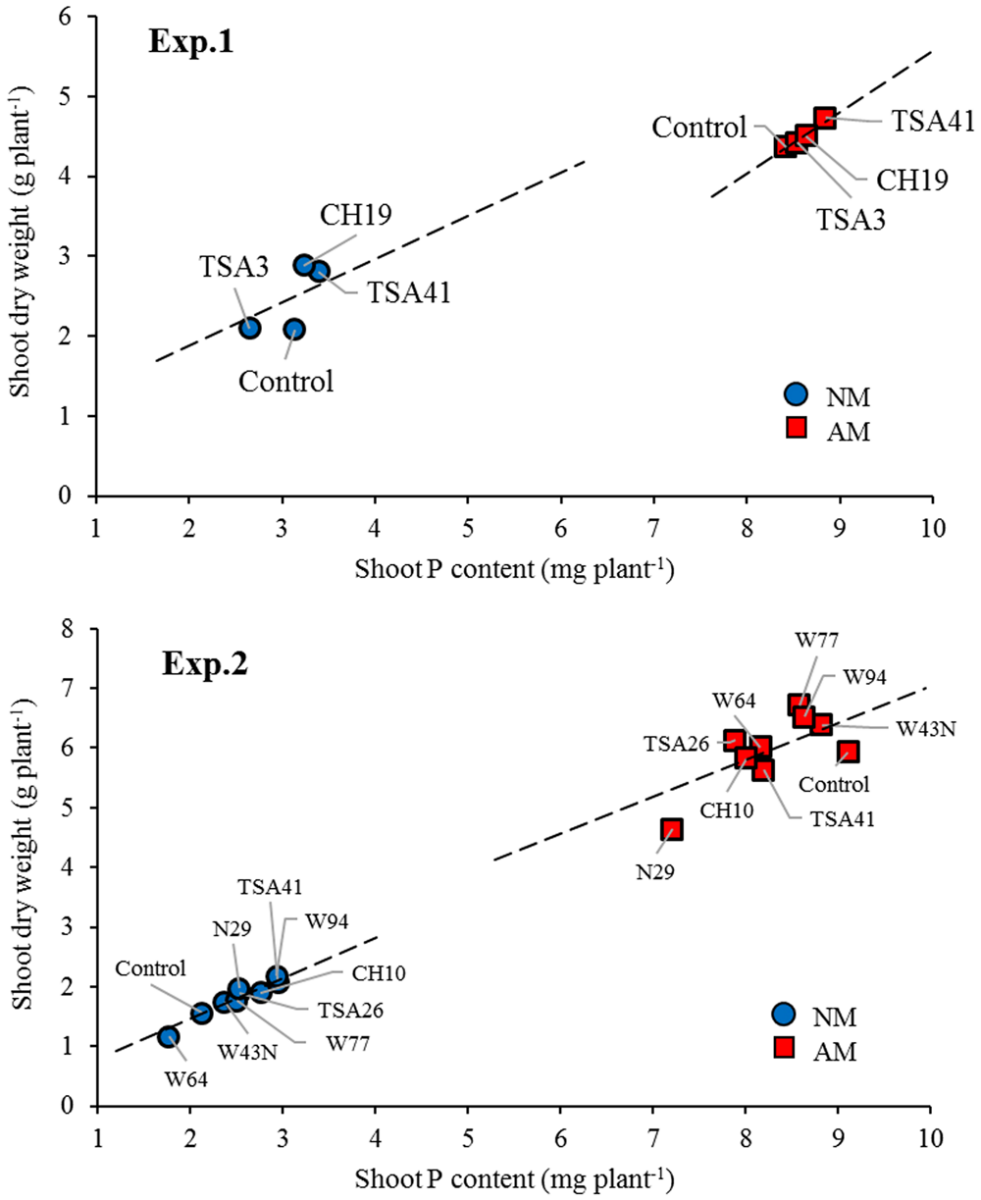
Linear relationship between shoot dry weight and shoot P content of mycorrhizal (AM) and non mycorrhizal (NM) *Z. mays* L. genotype Oh43 inoculated with different bacterial strains. Results of the regression analysis are as follows: Exp. 1, NM y = 0.542x + 0.796, R^2^ = 0.631; AMF y = 0.768x − 2.106, R² = 0.609; Exp. 2, NM, y = 0.678x + 0.111, R² = 0.835; AMF y = 0.619x + 0.851, R² = 0.483. For abbreviations see Table 1.

**Figure 3:**
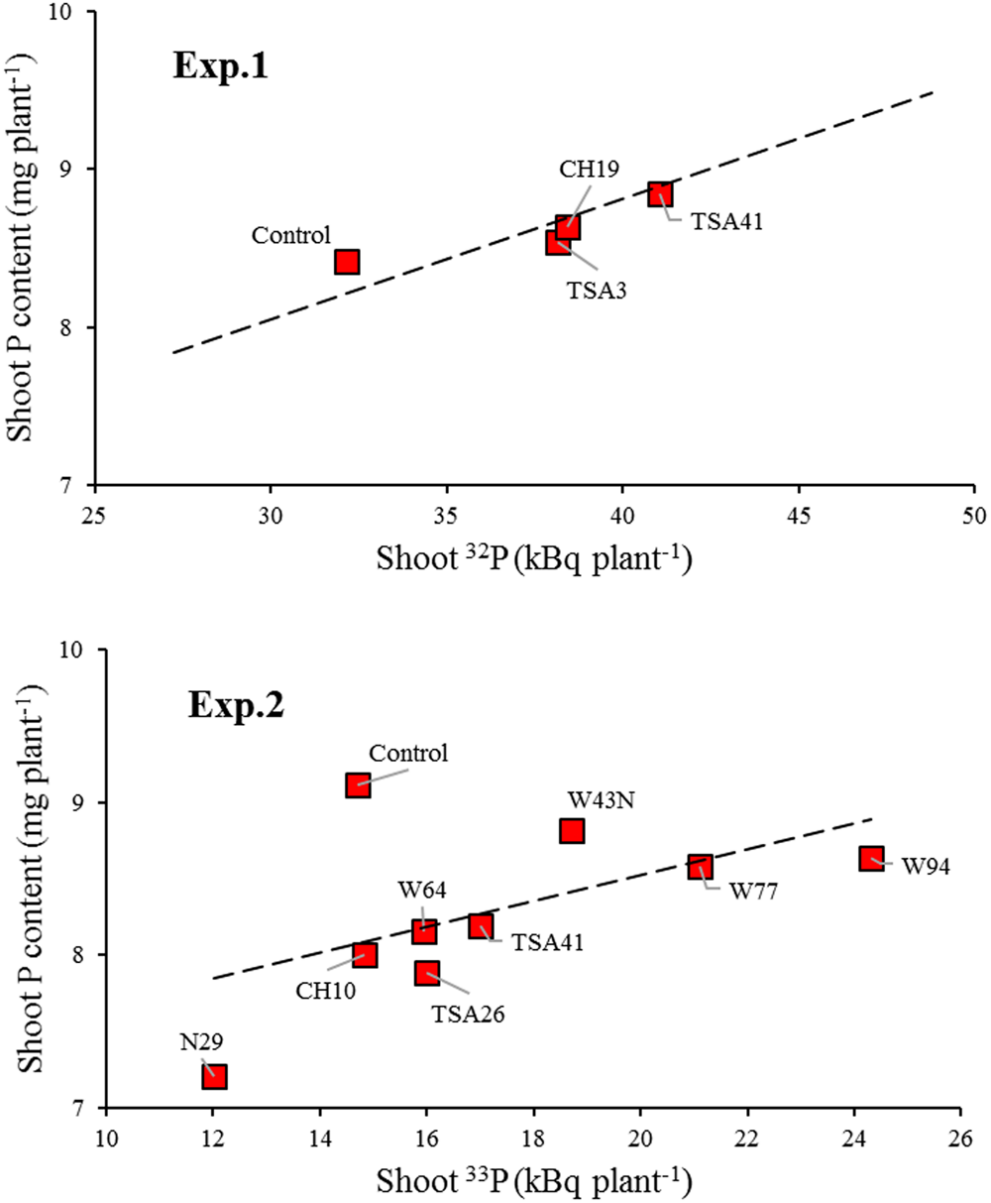
Linear relationship between total ^32^P/^33^P and total P content of mycorrhizal *Z. mays* L. genotype Oh43 inoculated with *R. irregularis* in combination with different bacterial strains. Result of the regression analysis is as follows: Exp. 1, y = 0.077x + 5.748, R² = 0.535; Exp. 2, y = 0.085x + 6.831, R² = 0.303. For abbreviations see Table 1.

In accordance with the P-limiting growth conditions, plant growth responses to mycorrhizal colonization overall mirrored the increases in shoot P contents (Fig. 2). Similar correlations between DW and P content in shoots were observed within each individual group of NM or AM-colonized plants in both experiments (Fig. 2). These correlations between shoot DW and shoot P content were significant in both NM (*P* < 0.001) and AMF treatments (*P* < 0.001) in the two experiments, suggesting that the consistent and strong effects of bacteria on maize shoot growth depend on their ability to improve plant P uptake. Shoot P content was positively correlated with ^32^P shoot content in Exp. 1 (*P* = 0.001) (Fig. 3), showing that the effect of bacterial inoculation on plant P uptake was actually related to its effect on the contribution of AMF hyphae to P uptake. A similar, but not significant (*P* = 0.126) correlation was observed for total P and ^33^P in shoots of Exp. 2.

## Discussion

This work provides the first evidence of the positive interaction among AMF and bacteria isolated from their spores, leading to increased abundance of root-external mycelium. In several cases, this resulted in increased growth and P uptake via mycorrhizal pathway in maize plants while a particularly efficient strain, *Streptomyces* sp. W94, facilitated P uptake by AMF hyphae from a root free soil compartment.

The marked AMF-related enhancement in P uptake and growth of the inbred maize line Oh43 is typical for plants grown under P-limiting conditions^10^ and the magnitude of the responses is in good agreement with previous results obtained with the same maize genotype^45, 46^. The key role of the root-external mycelium in AMF effects on plant P uptake was confirmed by the large uptake of ^32^P or ^33^P from the root free HC into shoots; this uptake corresponded to 12–15% and 4–9% of the radioactivity that had been added to and equilibrated with the native P pools in the HC soil in Exp. 1 and 2, respectively.

The differential effects of inoculated bacterial strains on plant performance were consistent with their previous functional characterization^43^. Hence, our findings that *Streptomyces* sp. W43N showed the higher values of root DW in AM plants and that *S. meliloti* TSA26, *Bacillus* sp. CH10, *Streptomyces* sp. W43N and *Streptomyces* sp. W94 increased root P content of NM plants are in agreement with their functional traits, as they showed the largest phosphate solubilisation and a high phytate mineralization^43^. Indeed, a growing number of PGPB, including *Bacillus* and rhizobia, have been reported to mobilize soil P from insoluble to soluble forms through two main processes: the secretion of organic anions (such as gluconic acid) chelating cations bound to phosphate and the production of phytase/phospathase enzymes able to hydrolyse organic forms of phosphate compounds^47–50^. Since P was the main growth limiting factor in our work, as evidenced by the low P concentration found in NM plants, approximately 1.3 mg g^−1^ and 1.4 mg g^−1^ of P in Exp. 1 and Exp. 2 respectively, the bacterial strains utilized here can be considered promising inoculants, providing a possible strategy to enhance P availability and root uptake.

In addition to their possible direct effects on P mobilization, the bacteria also influenced the AMF symbiosis. Hence, seven out of 10 bacterial strains significantly improved hyphal length growth and two of the strains, *S. meliloti* TSA41 and *Streptomyces* sp. W43N, increased hyphal length as much as 24% over the levels measured in AMF plants without bacterial inoculation. Such strains, belonging to the gram-negative Proteobacteria and gram-positive Firmicutes and Actinomycetes, could thus be classified as mycorrhiza helper bacteria (MHB) for their promotion of mycorrhizal functioning^51^. Previous studies showed that different species of *Streptomyces* stimulated spore germination of *G*. *margarita* through the production of volatile compounds^52^, that *Brevibacillus* sp. promoted mycorrhizal colonization by *F. mosseae* in *Trifolium pratense* ^53^ and that *Paenibacillus rhizosphaerae*, *Azospirillum* sp., and *Rhizobium etli* significantly increased the growth of extraradical hyphae and the number of newly formed spores of *R. irregularis* cultivated *in vitro* ^54^. Another recent study reported that several *Pseudomonas* strains could stimulate the *in vitro* growth of *R. irregularis* ERM^55^. Our data are particularly interesting, as they were obtained *in vivo*, by a method which, though labour intensive^13^, allows the quantification of the actual abundance of AMF hyphae in the soil. Bacterial enhancement of their growth is consistent with a previous study where inoculation of *P. putida* stimulated the growth of *Glomus fistulosum* extraradical hyphae, although AM colonization was not improved^56^. Similarly, *P. fluorescens* DF57 and *Burkolderia cepacia* Bc2 enhanced mycelial development of *G. caledonium* and *R. irregularis*, respectively^57, 58^. So far, the mechanisms underlying such outcome remain unknown. However, there is evidence to support the hypothesis that bacterial production of IAA and indole butyric acid (IBA) play roles in AMF hyphal elongation. For example, IAA-producing *Paenibacillus* strains increased *Glomus intraradices* development^59^ while the exogenous application of IBA promoted the ERM of *Diversispora versiformis* in association with trifoliate orange^60^.

In this work, plants inoculated with AMF, alone or in combination with bacteria, significantly enhanced root development. Specifically, in Exp. 2, root length of mycorrhizal maize plants was three fold the values for non-mycorrhizal plants. Interestingly, the root length in NM plants was increased up to 53% by inoculation with *Bacillus* sp. CH10. Such results are consistent with recent reports on the *in vitro* production of IAA by *Bacillus* sp. CH10^43^. Indole acetic acid is an important phytohormone, playing a key role in plant growth by affecting many functional activities, such as cell division, elongation, root initiation and fruit development^61^. So far, a number of strains belonging to different species, including several *Bacillus* species, have been reported to synthesize IAA. For instance, IAA producing *B. flexus* P4 and *Bacillus* sp. S6 increased *Solanum tuberosum* root length by 40 and 50%, respectively, compared with non-inoculated control^62^, while *S. meliloti* TSA41 and *Streptomyces* sp. W43N highly improved the levels of IAA in Dark Opal basil plants, in comparison with controls^63^.

Radioactive P tracers (^32^P and ^33^P) are widely applied to study soil P availability as well as P uptake from different soil and fertilizer P sources^64^. However, no studies have utilized isotopic techniques to assess the interactions between AMF and their strictly associated bacteria and to determine their contribution to plant P uptake, while only a few works investigated the effects of rhizobacteria on P nutrition^65–67^. Here, the labelling of HC soil with radioactive P (^32^P or ^33^P) made it possible to distinguish between P uptake by AM hyphae and P uptake by roots and hyphae in combination and to detect a wide variation among the bacterial strains in their ability to improve P uptake via mycorrhizal pathway. These findings are consistent with the monitoring data for shoot radioactivity, showing that AM plants had higher counts in the presence than in the absence of inoculated bacteria at 16, 18, 21 and 23 d after sowing. Interestingly, both shoot ^33^P and hyphal length specific ^33^P uptake was greater in AM plants inoculated with *Streptomyces* sp. W94, than in the corresponding no bacteria control, while *Streptomyces* sp. W77 enhanced hyphal length specific ^33^P uptake. In our work, the bacterial strains were applied directly to the seed and after 15 days to the seedlings instead to the hyphal compartment. As such bacteria may be associated not only to AMF spores, but also to plant roots, further studies could show whether their effects on plant growth and P uptake are maintained when inoculated far from the roots, for example in the root-free compartment.

The influence of bacterial inoculation on the AMF-mediated P uptake from the HC was further supported by the significant positive correlation between shoot P content and shoot ^32^P. These data suggest that the mechanism underlining the higher shoot ^33^P content of bacteria inoculated plants was less related to phosphate solubilization than to the bacterial induction of greater root length and/or hyphal abundance. Our results are consistent with previous findings obtained using isotopic-labeling technique, where *P. protegens* CHA0 did not enhance P release from soil, although it was able to solubilize P *in vitro* ^67^. A number of studies reported that *in vitro* P solubilization by microorganisms does not necessarily imply that inoculated plants will grow better. For instance, Taurian *et al*.^68^ screened 110 potential P solubilizing bacteria *in vitro* but only one of them promoted peanut growth. Thus, P mobilization by PGP bacteria is a complex phenomenon that can be ascribed to multiple functional traits differentially affecting plant growth. Indeed, a large array of bacteria belonging to different genera was reported to indirectly improve P uptake by altering root architecture through IAA production^69^.

In conclusion, root colonization by AMF produced large plant growth responses, while seven bacterial strains further facilitated root growth and P uptake by promoting the development of AMF extraradical mycelium. Among the tested strains, *Streptomyces* sp. W94 produced the largest increases in uptake and translocation of ^33^P, while *Streptomyces* sp. W77 highly enhanced hyphal length specific uptake of ^33^P. The positive relationship between AMF-mediated P absorption and shoot P content was significantly influenced by the bacteria inoculants and such results emphasize the potential importance of managing both AMF and their microbiota for improving P acquisition by crops. Our findings show that interactions among plants, AMF and mycorrhizospheric bacteria are complex and may produce different outcomes, depending on the composition of the microbial inoculum. A future focus on the interactions between different plant, AMF and bacteria genotypes, including effects of growth conditions will deepen our knowledge of the functioning of such multipartite interactions and help us to select the most efficient consortia to develop biofertilizers and bioenhancers for sustainable agriculture systems.

## Methods

### Experiment 1

The Exp. 1 was set up using compartmented pots with a root hyphal compartment (RHC) and a hyphal compartment (HC)^17, 70^. RHC was a 1.5 kg plastic pot lined with a plastic bag, while HC was a small plastic cylinder capped at both ends with a 25 µm nylon mesh. This mesh allowed fungal hyphae to pass through and absorb nutrients but prevented the ingrowth of roots from RHC. The growth medium used was 1:1 mixture of γ-irradiated soil (15 kGy) and autoclaved quartz sand. The soil was a local low P sandy loam and had the following characteristics: 10% clay, 12% silt, 46% fine sand, 30% coarse sand, 1.2% total Organic Carbon, 10 mg P kg^−1^ 0.5 M NaHCO_3_ extractable P^71^. In order to ensure an adequacy of all nutrients but P, the growth medium, hereafter called soil, was supplemented with basal nutrients minus P. Soil for the HC, was uniformly labelled with 5 kBq g^−1^ soil of carrier-free H_3_^32^PO_4_. The RHC was filled with 1445 g of unlabelled soil while the HC was filled with 55 g of ^32^P-labelled soil and placed horizontally at the same depth in all pots.

Uniformly sized seeds of *Zea mays* L., inbred line Oh43, were surface sterilized twice in 1.5% (v/v) sodium hypochlorite solution for 10 min and rinsed thoroughly with sterile MilliQ water. The seeds were imbibed 2 h in sterile water and pre-germinated on moist paper in Petri dishes kept in darkness for 2 days at 27 °C until radicles emerged. The germinated seeds were then sown, one per 1.5 kg pot, at approximately 2 cm depth.

Half of the pots were inoculated with the AM fungus *R. irregularis* Schenck and Smith BEG 87 that had been propagated in pot cultures with *Trifolium subterraneum* L. for four months in the same soil as described above. The trap plants were well colonized and had produced high quantities of spores and extraradical mycelium. Pot cultures were air dried, shoots were removed and a crude inoculum was prepared by mixing the soil with chopped mycorrhizal roots, extraradical mycelium, and spores and thereafter stored at 4 °C. Ten per cent (w/w) of this crude inoculum was mixed into the RHC soil. RHCs of non-mycorrhizal plants received a mock inoculum produced by sterilizing the appropriate amount of mycorrhizal inoculum. All pots, non-mycorrhizal and mycorrhizal, received 20 mL of soil filtrate (through Whatman paper), made by dissolving 200 g of BEG 87 inoculum in 1500 mL water, to ensure a common microbiota to all treatments.

Three bacterial strains, *S. meliloti* TSA3, *S. meliloti* TSA41 and *Lysinibacillus fusiformis* CH19 were used to inoculate the plants, alone or in combination with the mycorrhizal inoculum. The bacteria were previously isolated from *R. irregularis* IMA6 (formerly *G. intraradices*)^43^ spores and were selected for their plant growth promoting (PGP) properties, such as the production of indole acetic acid (IAA) and siderophores, phosphatase-solubilizing and phytase-mineralizing activities. Each bacterial strain was cultured and inoculum prepared as previously reported^72^. For each strain, bacterial density of the suspension was assessed using a Thoma cell chamber to obtain a final cell concentration of 10^9^ cells mL^−1^. At sowing, inoculation was performed by adding on the surface of sterilized seeds 1.5 mL of 10^9^ cells mL^−1^. Ten days after sowing, seedlings were inoculated with the same amount of bacterial suspension. The same amount of physiological saline solution was provided to uninoculated plants.

The experiment combined mycorrhizal and bacterial inoculation. The four AMF treatments were: inoculation with *R. irregularis* and inoculation with the bacterial strains *S. meliloti* TSA3, *S. meliloti* TSA41, *L. fusiformis* CH19. The corresponding non-AMF treatments were set up. Each of the resulting eight treatments had five biological replicates.

Plants were grown in a controlled environment chamber at the Technical University of Denmark, Risø Campus, under a day/night cycle of 14/10 h, 25/20 °C, 60% relative humidity and an average light intensity of 500 μmol m^−2^ sec^−1^. Plants were watered by weight to 60% of water-holding capacity over the course of the experiment and nitrogen (as NH_4_NO_3_) was added to the pots 16, 19, and 22 days after sowing, providing a total of 195 mg N per pot. Uptake of ^32^P from HC compartments was monitored by a G-M tube (Mini 900 Meter, Thermo Fisher Scientific, Waltham, Ma, USA) on the middle part of the youngest fully developed leaf at 1–3 days intervals between 13 and 28 days after planting.

### Experiment 2

The set up and growth conditions of Exp. 2 were similar to those of Exp. 1, except that nine bacterial treatments were included: inoculation with *S. meliloti* TSA41, *S. meliloti* TSA26, *Streptomyces* sp. W43N, *Streptomyces* sp. W64, *Streptomyces* sp. W77, *Streptomyces* sp. W94, *S. meliloti* N29, *Bacillus* sp. *pumilus* group CH10 or no inoculation. As for Exp. 1, each treatment had five biological replicates. In Exp. 2, the HC soil was labelled with 5 kBq g^−1^ soil of carrier-free H_3_^33^PO_4_ and plants were maintained in a growth chamber at University of Copenhagen.

### Harvest and samples analysis

Harvest procedures and measurements were similar in the two experiments. Plant height and stem diameter (only in Exp. 1) were measured just before harvest, 30 days after sowing. Shoots were cut at soil surface level and weighed and HCs were removed from the pots and stored frozen. Soil from the HCs was dried at 50 °C for 24 h and used for the determination of hyphal length density (HLD). HLD was measured following the membrane filter procedure described in Jakobsen *et al*.^13^.

Roots were carefully washed free of soil, blotted and total fresh weight was determined. A weighed representative subsample for assessment of AM colonization was stored in 50% ethanol. Dry weights of shoots and roots were recorded after 48 h drying at 70 °C in a ventilated oven. Shoot and root tissues were ground to powder and wet oxidised in a solution of nitric and perchloric acids (4:1, v-v). Digests were analysed for P concentration by the molybdenum blue method (Murphy and Riley, 1962) using AutoAnalyzer 3 (Bran + Luebbe, Norderstedt, Germany) for Exp. 1 and FiaStar 5000 Analyzer (Foss Analytical AB, Höganäs, Sweden) for Exp. 2. The radioactivity (^32^P or ^33^P) in the digests was determined by liquid scintillation counting using the scintillation mix Ultima Gold ™ with TriCarb 1900 TR for Exp. 1 and TriCarb 2910 TR for Exp. 2 (Perkin Elmer, Waltham, MA, USA); counts were corrected for decay and background. Radioactivity and P concentration was used to calculate the specific activity (SA) in the shoots (^32^P/^31^P or ^33^P/^31^P).

Percentages of AMF colonization and total root length were assessed under a dissecting microscope using the gridline intersect method^73^ after clearing of roots in 10% KOH and staining with Trypan blue in lactic acid (0.05%).

### Statistical analysis

Statistical analysis was performed using SPSS 20.0 software (IBM Corp., Armon, NY Inc, USA). Data were analysed by one-way ANOVA, using treatment as factor, followed by Fisher’s least significant difference (LSD) with cut-off significance at *P* < 0.05. Data were transformed as needed to obtain normality and homogeneity of variances. If a normal distribution and/or homogeneity of data could not be guaranteed, we performed a Student’s *t* test to compare means between NM and AMF plants. The means within each mycorrhizal treatment were then analysed by one-way ANOVA followed by LSD or Dunnett’s T3 post-hoc test (where equal variances were not assumed). Correlations among shoot biomass, P content, P uptake and HDL were examined by linear regression analysis (*P* < 0.05). In Exp. 1 four seedlings that died were treated as missing values whilst in Exp. 2 two extreme outliers and two plants with abnormal growth were excluded from all analyses. This resulted in 36 and 86 pots in Exp. 1 and Exp. 2, respectively.
